# Case Report: A Case of Acute T Lymphoblastic Leukemia With Mixed Infection of Lethal Invasive Mucormycosis and Multi-Drug Resistant Bacteria

**DOI:** 10.3389/fmed.2022.854338

**Published:** 2022-04-11

**Authors:** Qingya Cui, Haiping Dai, Depei Wu, Jun He, Yang Xu, Xiaowen Tang, Jie Xu

**Affiliations:** ^1^National Clinical Research Center for Hematologic Diseases, Hematology Department, Jiangsu Institute of Hematology, The First Hospital Affiliated to Soochow University, Suzhou, China; ^2^Dinfectome Inc., Nanjing, China; ^3^Center of Clinical Laboratory, The First Affiliated Hospital of Soochow University, Suzhou, China

**Keywords:** pulmonary mucormycosis, multi-drug resistant bacteria, Mucor infection, metagenomic next-generation sequencing, acute T lymphoblastic leukemia

## Abstract

Pulmonary mucormycosis (PM) is a rare and life-threatening fungal infection. Here, we report a case of an acute T lymphoblastic leukemia patient with mixed infections of lethal invasive Mucormycosis and multi-drug resistant (MDR) bacteria. After receiving anti-infection drugs to control the patient's fever, he was treated with induction chemotherapy. However, the malignant hematological disease was poorly controlled by the chemotherapy and the patient developed more symptoms of infection. Although the results of multiple β-D-Glucan (G) and Galactomannan (GM) tests remained negative, several pathogens were detected using metagenomic next-generation sequencing (mNGS). In particular, mNGS identified *Malassezia pachydermum, Mucor racemosus*, and *Lauteria mirabilis* in the peripheral blood and local secretion samples. The Mucor and bacterial infections were further confirmed via multi-site and repeated fungal and bacterial cultures, respectively. Despite adjusting the anti-infection therapy according to the diagnostic results, the patient's blood disease and symptoms of infection were not alleviated. Additionally, the MDR *Acinetobacter baumannii* infection/colonization was not confirmed until the seventh culture of the peripheral venous catheter tip. Due to the patient's deteriorating conditions, his family decided to withdraw him from further treatment. Overall, mNGS can facilitate a diagnosis of Mucormycosis by providing clinical and therapeutic information to support conventional diagnostic approaches. For the early and timely diagnosis and treatment of PM, it is also necessary to consider the malignant hematological conditions and repeated tests through multiple detection methods.

## Introduction

Pulmonary mucormycosis (PM) is caused by uncommon fungal infections and its mortality rate is between 30 and 65%, with an average survival time of only 27 days (1, 2). Despite the importance of the early diagnosis and treatment of PM, the detection of PM is challenging due to the lack of specific clinical manifestations. In particular, PM can occur with either the presence or absence of suppurative inflammation, and some common pathological manifestations of PM include the invasion of fungi in bronchus and lung tissue, the coagulative necrosis of the lungs, pulmonary hemorrhage, vascular invasion-induced thrombosis, hemorrhagic pulmonary infarction, and hematogenous dissemination (3). The imaging manifestations and the dynamic changes during PM are similar to invasive pulmonary aspergillosis (IPA), and are relatively complex, thus, further complicating diagnosis (4, 5).

Voriconazole is generally ineffective as a preventive treatment for PM, and PM patients generally experience massive hemoptysis and tend to be negative in β-D-Glucan and Galactomannan (G and GM) tests (6, 7). PM patients are likely to exhibit additional clinical features, including pulmonary lesions with sinusitis (e.g., bone destruction), halo sign, multifocal pneumonia, and pleural effusion (8).

In this study, we report a case of an acute T-cell lymphoblastic leukemia patient who experienced mixed infections of Mucormycosis and multi-drug resistant (MDR) bacteria. The case provides potential insights into the diagnosis and treatment of PM patients with complex disease conditions.

## Case Description and Diagnostic Assessment

A male patient was admitted to hospital on November 21, 2018 because of pain in the right rib that had lasted for 1 week, and fever that had been present for 1 day. The patient was working in a tea stir-frying company and had no previous medical, family, or psychosocial history. The patient's routine blood examination revealed a hemoglobin content (Hb) of 84 g/L, white blood cell (WBC) count of 64.18 × 10^9^/L, a neutrophil count of 0.3 × 10^9^/L, and a platelet count (PLT) of 75 × 10^9^/L. The patient's bone marrow morphology and immunology results suggested that he had acute T-cell lymphoblastic leukemia. Specifically, a chest computed tomography (CT) scan revealed a soft tissue shadow at the anterior superior mediastinum, as well as lymph node enlargement and splenomegaly. Bone marrow cell morphology revealed significantly active hyperplasia, 91% of which was due to primitive and immature cells. By analyzing 92.3% of the immature cell population, bone marrow immunostaining was positive for CD7, CD34, CD10, CD56, cCD3, and CD99, and weakly-positive for CD13, CD38, and T lymphocyte expression, which was consistent with the immunostaining data for early T-cell progenitors (ETPs). According to genetic analyses, the karyotype of the patient was 46,XY,del(11)(p11),del(17)(p11)[6]/46,idem,der(1)[4]. Upon examining 43 leukemia-related fusion genes by multiplex PCR, no fusion transcripts were detected. Additionally, the copy number of *WT1* was 1,322 copies/10,000 copies of *ABL*. However, mutations in *SF3B1, NOTCH1, PHF6, SUZ12, SUZ12p, GATA3*, and *CTCF* were detected by next-generation sequencing (NGS).

**Figure 1:**
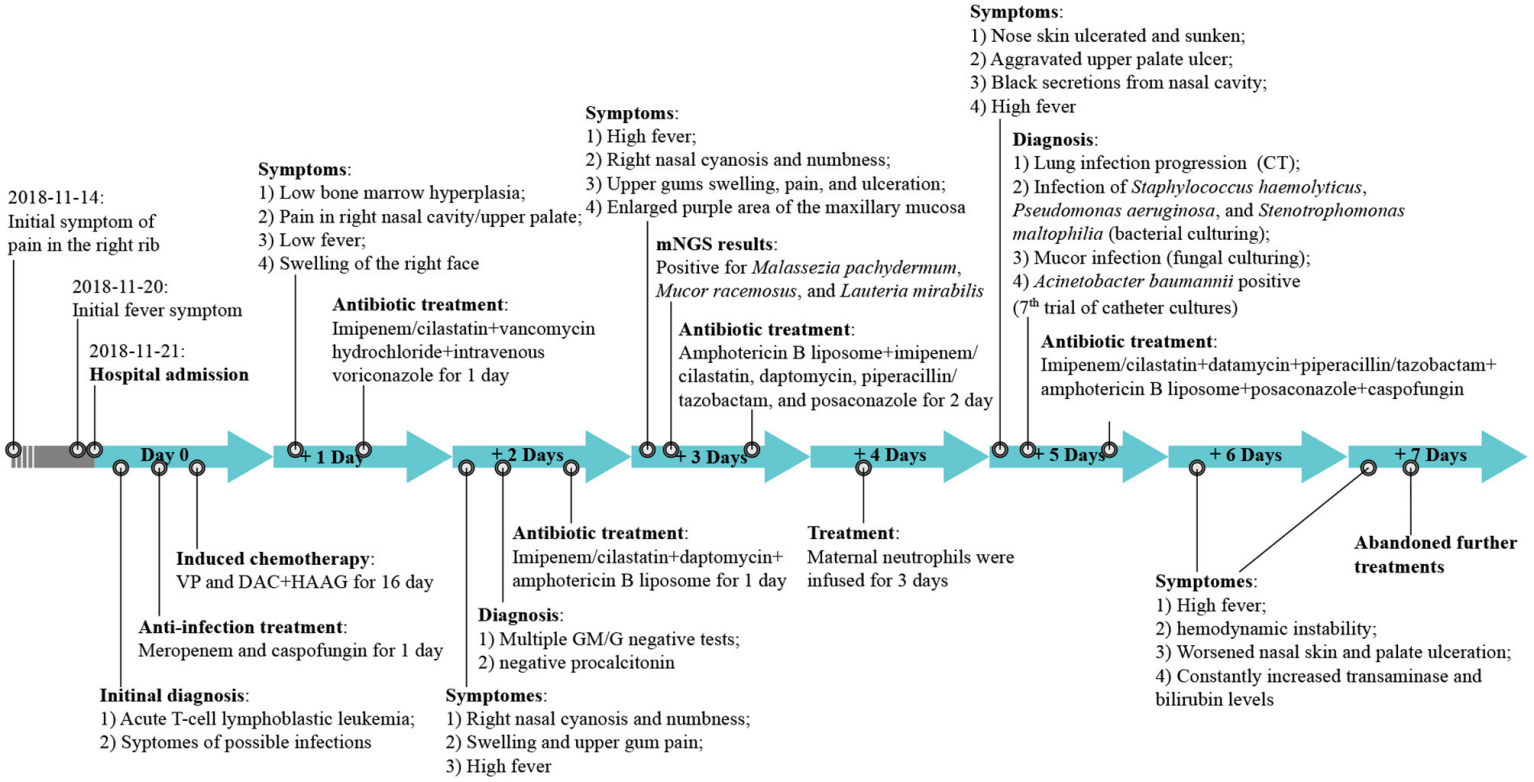
The treatment timeline. VP, Vincristine + Prednisone; DAC, decitabine; HAAG, homoharringtonine (H), cytarabine (A), aclarubicin (A), granulocyte colony stimulating factor (G-CSF); CT, computed tomography; GM/M, β-D-Glucan (G); Galactomannan (GM) tests; mNGS, metagenomic next-generation sequencing.

**Figure 2:**
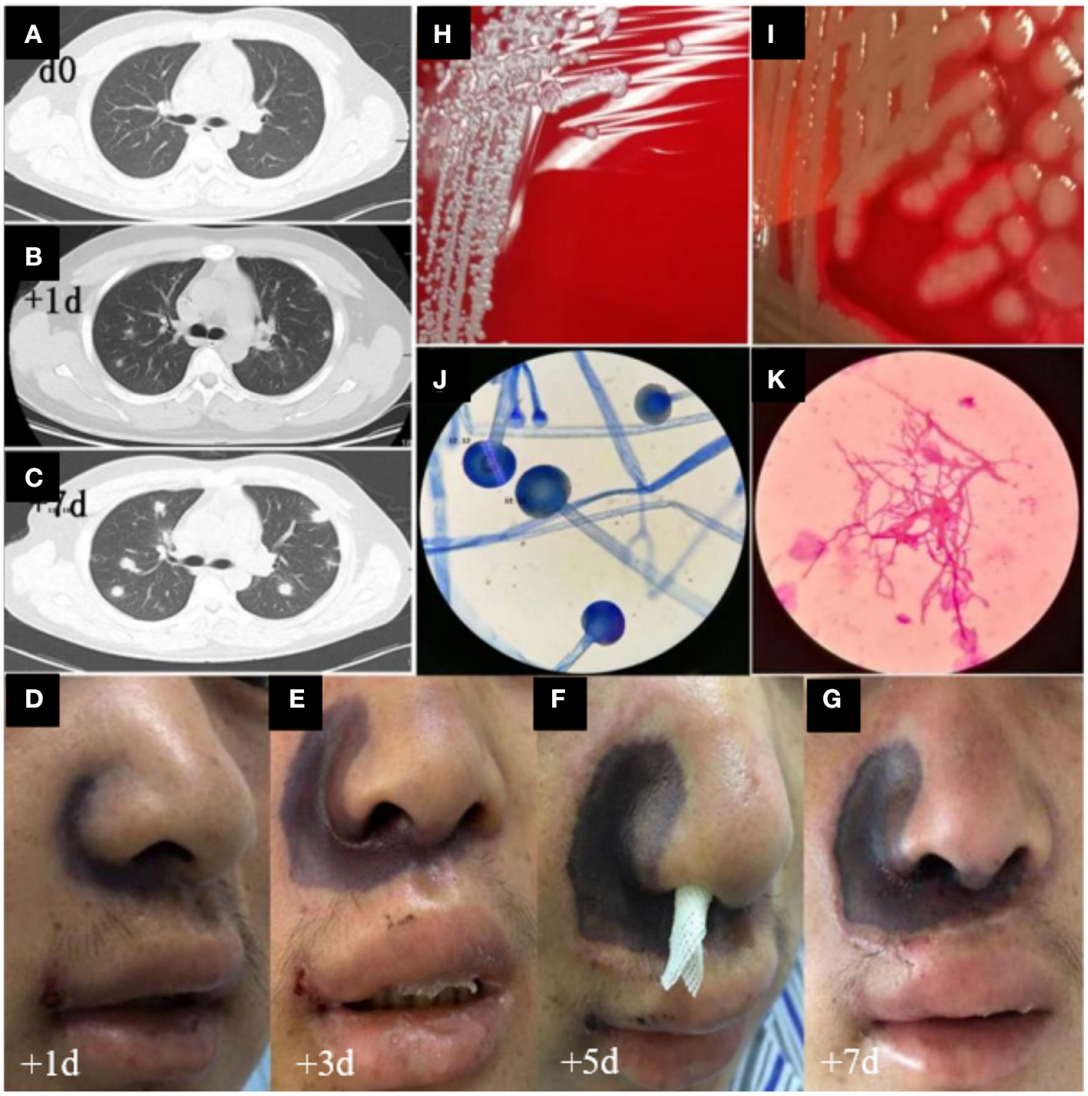
CT images, advancement of skin damage, and morphologic features of microbiological cultures in this case. **(A–C)** Day 0, day 1, and day 7 of the chest CT images, respectively. **(D–G)** Day 1, 3, 5, and 7 images of the nasal skin, respectively. **(H,I)** Bacterial cultures of *Staphylococcus haemolyticus* and MDR *Pseudomonas aeruginosa*, respectively. **(J,K)** The morphologic features of Mucor.

Based on the patient's neutrophil counts, which suggested agranulocytosis, anti-infection treatment with meropenem and caspofungin was administered. VP (Vincristine + Prednisone) combined with decitabine (DAC; 20 mg/m^2^, days 1–5) plus HAAG regimen-based chemotherapy (homoharringtonine (H) 1 mg/d, days 3–16; cytarabine (A) 10 mg/m^2^, injected subcutaneously every 12 h, days 3–16; aclarubicin (A) 10 mg/d, days 3–10; granulocyte colony stimulating factor (G-CSF) 50-600 μg/day, days 2–9 unless WBC count was higher than 20 × 10^9^/L) was administered after controlling the fever (Figure 1). By re-examining the bone marrow cell morphology on the 1st day of chemotherapy, the result revealed low bone marrow hyperplasia, 91% of which were due to primitive and immature cells. In addition, the patient experienced pain in the right nasal cavity and upper palate, together with a low fever and swelling of the right side of the face. The patient's antibiotic therapy was therefore adjusted to imipenem/cilastatin, vancomycin hydrochloride, and intravenous voriconazole. Twenty-four hours later, the patient's skin on the right nasal wing was cyanotic with numbness, his swelling and the upper gum pain became more severe, and he developed a continuously high fever. The patient was negative for procalcitonin and the results of the GM and G test remained negative after multiple trials. The patient's cranial CT revealed foreign bodies in the nasal cavity and maxillary sinusitis, while endoscopic tests revealed nasal suppurative infection. The therapy was then immediately adjusted to imipenem/cilastatin, daptomycin, amphotericin B liposome, and local douche (Figure 1), and peripheral blood and local secretion were collected for metagenomic next-generation sequencing (mNGS). On the 3rd day, the chest CT revealed nodular lesions (Figures 2A–C), while the patient continued to suffer from continuous high fever. The numbness of the skin on the right nasal wing remained and the right nasal cyanosis became enlarged and darker (Figures 2D,E). The upper gums exhibited obvious swelling, pain, and ulceration, and the purple area of the maxillary mucosa expanded, which was accompanied by ulceration.

Based on the mNGS results, the patient was positive for the presence of *Malassezia pachydermum, Mucor racemosus*, and *Lauteria mirabilis*. The antibiotic therapy was then adjusted to amphotericin B liposome, imipenem/cilastatin, daptomycin, piperacillin/tazobactam, and posaconazole (Figure 1). On the 4th day, we infused the maternal neutrophils for 3 days (Figure 1). On the 5th day, the skin of the nose was ulcerated and sunken, which was not alleviated during the course of the treatment (Figures 2F,G). Additionally, the ulcer on the upper palate was aggravated, and the nasal cavity displayed black secretions. The chest CT consistently indicated the progression of the lung infection. Bacterial cultures of the nasal secretions indicated the presence of *Staphylococcus haemolyticus*, MDR *Pseudomonas aeruginosa*, and *Stenotrophomonas maltophilia* (Figures 2H,I), while fungal cultures suggested Mucor infection after multiple cultures (Figures 2J,K). The results of the first six catheter cultures of the peripheral blood were all negative, and it was not until the 7th culture (on the 5th day) that MDR *Acinetobacter baumannii* was detected. The antibiotic therapy was then adjusted to imipenem/cilastatin, datamycin, piperacillin/tazobactam, amphotericin B liposome, posaconazole, and caspofungin (Figure 1). Nevertheless, the patient experienced a sustained high fever, hemodynamic instability, continuously worsening nasal skin and palate ulcerations, and exhibited increasing transaminase and bilirubin levels. Due to the deteriorating conditions, the patient's family decided to withdraw him from further treatment. The patient failed to achieve remission after 16 days of induction chemotherapy, and experienced serious infections and agranulocytosis after chemotherapy. The patient died the day after discharge, on day 16.

## Discussion

PM is a severe and deadly disease. According to Chamilos et al., lung disease patients who had sinusitis and did not responded to preventive treatment with voriconazole were more likely to have PM (9). Additionally, multiple lesions (≥10 nodules) and pleural effusion were the two major independent predictors of PM (9). In our case, the patient was positive for ETP with a poor prognosis, and induction chemotherapy did not alleviate the symptoms. The patient also had nasal sinus infections, multiple negative GM and G results, and an insignificant increase of procalcitonin (PCT) levels, which was consistent with the criteria of PM put forth by Chamilos et al. The culture results of the local secretion confirmed the presence of mucormycosis infection in the nasal sinus, which implied that the pulmonary infection might have resulted from the pathogen spreading via the airway.

Although we adjusted the antifungal therapy (i.e., amphotericin B liposome, posaconazole and caspofungin, and donor neutrophil infusion), the patient's blood disease was not alleviated. Furthermore, he continued to suffer from persistent severe agranulocytosis, uncontrolled local infection, and the spread of pulmonary lesions. The results of the 7th blood culture revealed the presence of MDR *Acinetobacter baumannii*, thus, indicating a worse and more fatal disease condition than previously expected.

Several clinical tests are needed to confirm Mucormycosis infections, including histopathology, direct examinations, tissue culture, and the testing of respiratory secretions and bronchoalveolar lavage fluid. Conventional evaluation techniques typically have limited sensitivity and specificity, and bacterial/fungal cultures often give negative results, despite positive microscopic examinations (7). Indeed, cultures can only detect ~50% of cases of Mucormycosis infection (10). Recent advancements in polymerase chain reaction (PCR) technology have contributed to the development of rapid, accurate, and sensitive methods for pathogen detection. However, some PCR assays cannot provide such information due to their limit of detection, sensitivity, specificity, and cross-reactivity. Given that many such PCR assays lack clinical validation and internal evaluation, their applications are mainly restricted to research purposes (11).

mNGS is a high-throughput technology that provides direct information about the type of infection, without relying on microbial cultures (12). Considering the rapid speed and high sensitivity of mNGS, this technology may facilitate the timely diagnosis of disease, especially in life-threatening scenarios. In the present case, mNGS detected *Mucor racemosus* infection on day 3, whereas it was not until day 5 that fungal culture confirmed the Mucor infection. As a result, mNGS demonstrated its substantial clinical potential in facilitating diagnosis. However, as some pathogens identified by mNGS are opportunistic and rarely lead to infection, it remains necessary to verify the infectious agent via culture tests. Thus, mNGS can be used to guide clinical laboratories to adjust the culture conditions for fastidious or specific microorganisms, which may increase diagnostic and prognostic accuracy, and improve treatment efficacy.

The unfavorable outcomes of the current PM case revealed several clinical implications: (1) One of the major reasons for the failure of the anti-PM therapy was due to the uncontrolled malignant hematological disease. (2) The patient's occupational environment imparted potential risks for the long-term exposure to molds, which might have led to the Mucor infection; however, we did not sufficiently consider the link between his disease and his working environment. (3) To determine the etiological basis for PM treatment, multi-site fungal and bacterial cultures, as well as mNGS may be necessary in patients with nasopharyngeal infections. (4) In cases of poorly-controlled nasopharyngeal infections after chemotherapy, the PM treatment should be adjusted in a timely manner. (5) Particular focus should be given to PM patients with severe agranulocytosis, as their disease could progress much faster than patients with non-malignant hematological diseases.

In conclusion, we reported a case of an acute T lymphoblastic leukemia patient with mixed infections of lethal invasive Mucormycosis and MDR bacteria. The patient experienced a poor clinical outcome, which indicated the importance of considering the malignant hematological disease conditions and patients' working/living environments. This case also highlighted the need to employ multiple diagnostic assays in cases of PM. mNGS may also facilitate the diagnosis of Mucormycosis by providing additional details in support of conventional diagnostic approaches.

## Data Availability Statement

The original contributions presented in the study are included in the article/supplementary material, further inquiries can be directed to the corresponding author/s.

## Ethics Statement

The studies involving human participants were reviewed and approved by the First Hospital Affiliated to Soochow University. The patients/participants provided their written informed consent to participate in this study. Written informed consent was obtained from the patient's family for the publication of this study.

## Author Contributions

XT and JX conceived and supervised the study. QC, HD, DW, JH, XT, and JX acquired the clinical data and performed patient follow-up. QC, HD, DW, JH, and YX acquired patient samples and performed the clinical testing. All authors participated in data analysis and interpretation, and were involved in manuscript preparation. All authors approved the final manuscript.

## Funding

The work was supported by the Jiangsu Provincial Key Research and Development Program (BE2019656).

## Conflict of Interest

YX is the employee of Dinfectome Inc. The remaining authors declare that the research was conducted in the absence of any commercial or financial relationships that could be construed as a potential conflict of interest.

## Publisher's Note

All claims expressed in this article are solely those of the authors and do not necessarily represent those of their affiliated organizations, or those of the publisher, the editors and the reviewers. Any product that may be evaluated in this article, or claim that may be made by its manufacturer, is not guaranteed or endorsed by the publisher.
